# Socioeconomic and Environmental Impacts of *Eucalyptus* Plantations in Ethiopia: An Evaluation of Benefits, Challenges, and Sustainable Practices

**DOI:** 10.1155/tswj/1780293

**Published:** 2025-01-12

**Authors:** Kiros Getachew Belachew, Wondwosson Kibrie Minale

**Affiliations:** ^1^Department of Natural Resource Management, College of Agriculture and Natural Resource, Debre Markos University, P. O. Box 269, Debre Markos, Amhara, Ethiopia; ^2^Department of Forestry and Climate Change, Injibara University, Injbara, Ethiopia

**Keywords:** deforestation, environmental impacts, *Eucalyptus* plantation, indigenous and exotic tree species

## Abstract

*Eucalyptus* was first introduced to Ethiopia in the late 19^th^ century to address the scarcity of firewood and construction wood in the capital city. Since then, it has spread across the country and has become an important source of income for many households while also reducing the need for deforestation. Despite concerns raised by environmentalists about its eco-hydrological impact, the plantation has expanded to cover a vast area of the nation, including farmlands and mountainous regions. Currently, around 506,000 hectares of land in Ethiopia are covered by *Eucalyptus* plantations. The growth of *Eucalyptus* plantations can be attributed to various socioeconomic, ecological, and biological factors, including the increasing demand for wood and wood products. However, this growth has also led to negative environmental consequences such as reduced surface and groundwater flow, decreased crop productivity, soil fertility degradation and depletion, and high water consumption, which can result in water scarcity. To address these environmental impacts, it is essential to select appropriate species and sites and implement proper silvicultural and land use planning before planting. Additionally, promoting renewable energy sources and planting environmentally sound fast-growing indigenous and exotic tree species can help reduce the adverse effects of *Eucalyptus* on the environment.

## 1. Introduction

In Ethiopia, approximately 80,000 hectares of natural forests have been cleared and converted into farmlands. Of this, 62.5% of the woodland natural forest area was cleared primarily for charcoal production, while the remaining 37.5% comprised bushlands cleared for fuelwood production [1]. The increasing demand for wood in industries, construction, and as fuel, driven by the country's rapidly growing population, has led to the widespread planting of fast-growing multipurpose exotic tree species, particularly *Eucalyptus* [2].

In the 1980s, the Ethiopian government introduced the planting of fast-growing *Eucalyptus* tree species as part of re-greening and plantation programs. These programs have gained significant recognition among policymakers as a means to increase the supply of forest products [3]. However, despite these efforts, tree planting only meets 6% of the biomass energy demand [2], therefore prompting farmers and foresters across Ethiopia to plant *Eucalyptus* trees extensively to satisfy the demand for wood biomass energy.

Today, Ethiopia has the largest area of *Eucalyptus* plantations in East Africa, covering approximately 506,000 ha of land [4]. Of the 133,041 ha of community tree plantations established between 1978 and 1989, 58% consist of *Eucalyptus* [5]. *Eucalyptus* is one of the most important planted tree species in Ethiopia, with research and reports indicating a rapid expansion due to its high socioeconomic value [6].

*Eucalyptus* trees, while often valued for their fast growth and utility in timber and paper products, can have significant environmental impacts, such as adversely affecting soil moisture and nutrient levels, which in turn can harm adjacent crops. This is significantly observed in the Amhara Region [7]. In response, various mitigation strategies have been proposed and implemented. One effective approach is the establishment of buffer zones, where native or less water-demanding species are planted between *Eucalyptus* plantations and agricultural lands to protect soil and water resources [8]. Additionally, promoting agroforestry practices, where *Eucalyptus* is integrated with other crops in a complementary manner, can help balance ecological needs and reduce competition for resources [9]. Sustainable management practices, such as controlling the density of *Eucalyptus* plantations and regularly monitoring soil health, are also critical in minimizing negative impacts [8]. These strategies aim to balance the benefits of *Eucalyptus* cultivation with the preservation of surrounding ecosystems and agricultural productivity.

*Eucalyptus* tree plantations have continued to be established on fertile agricultural lands each year, despite ecological concerns. According to a study by Alemayehu and Melka [10], 88% of *Eucalyptus* woodlots were planted on fertile lands due to their higher economic benefits compared to annual crops. Despite awareness of these negative impacts among policymakers, users, foresters, scientists, and farmers, the focus remains on the benefits, which are often seen to outweigh the drawbacks. Therefore, the primary objective of this review is to evaluate the extent of *Eucalyptus* plantation expansion and socioeconomic and its environmental impact in Ethiopia.

## 2. Methodology

This review article is grounded in a comprehensive analysis of related literature, sourced from various sources. Data were collected through a thorough review of scholarly articles, reports, books, online resources, and expert opinions. Over a hundred journal articles were gathered from Google Scholar, Scopus, and Web of Science and were organized into a personal Endnote library database. Additional references were identified by examining the bibliographies cited in the retrieved literature. From the total number of documents searched, thirty-three publications that focused on the impact of *Eucalyptus* plantation expansion on the environment were selected for a detailed review. To select the publications for detailed review, criteria included relevance to expansion and impact of *Eucalyptus* in Ethiopia and recency. These criteria ensured a well-rounded perspective on *Eucalyptus* impacts, recent findings, and geographical insights. The search for relevant literature was conducted using keyword selections related to the global and East African distribution of *Eucalyptus* the historical and current trends of *Eucalyptus* plantations in Ethiopia, the socioeconomic and environmental roles of *Eucalyptus* trees in Ethiopia, the causes of *Eucalyptus* plantation expansion in Ethiopia, and the environmental impact of *Eucalyptus* plantations in Ethiopia. Tables were prepared by summarizing the outputs of selected publications.

## 3. Literature Review

### 3.1. Origin, Nature, and Role of Eucalyptus Tree

#### 3.1.1. Origin and Nature of Eucalyptus Tree

*Eucalyptus* is a type of evergreen tree belonging to the Myrtaceae family, native to Australia, Indonesia, and the Philippines [11]. There are over 900 species of *Eucalyptus* but only about 100 are economically significant [12]. The tree is widely cultivated for its wood, paper pulp, gum, and medicinal oil. It was first introduced to Portugal four centuries ago and has since become widely distributed throughout Europe, Latin America, Asia, and Africa.

*Eucalyptus* provides numerous benefits to various countries, including fuelwood, charcoal, poles, posts, essential oils, and paper and pulp for manufacturing. Additionally, it offers nectar for bees, shade for animals and humans, and serves as a windbreak [13].

The name “*Eucalyptus*” is derived from two Greek words, “eu” meaning “well” and “kalyptos” meaning “hidden” or “well-covered.” The name was given by French botanist Jacques-Julien Houton de La Billardière, who categorized and named the species in 1799. The evergreen species of *Eucalyptus* are distinct from shrubs and multi-stemmed trees in height, growing up to 60 m tall and thriving at altitudes above 1850 m above sea level [14].

#### 3.1.2. Role of Eucalyptus Tree in Ethiopia

##### 3.1.2.1. Socioeconomic Roles of Eucalyptus Tree in Ethiopia

Land use changes are primarily influenced by socioeconomic, environmental, and cultural factors, as well as local livelihoods, policies, regulations, and cultural norms [15]. In Southern Ethiopia, the shift toward monoculture production was mainly driven by the economic benefits of crops like Chat (*Catha edulis*) and *Eucalyptus* [16]. Farmers are increasingly planting *Eucalyptus* trees to boost their household income, with the potential to earn up to half of their total income from this crop [17].

*Eucalyptus* is widely planted due to its ability to meet the growing demand for fuelwood and building materials, as well as its various other uses [18]. For example, in south central Ethiopia, *Eucalyptus* trees provide over 100% of the construction wood, 20% of the charcoal, and 93% of other wood products [19]. A similar investigation conducted in the Lake Plain area found that *Eucalyptus* was used primarily for fuel wood, income generation, and construction, with little regard for environmental conservation [20]. *Eucalyptus* also offers medicinal benefits, such as treating common colds, flu, and fever [17].

Several studies conducted in Ethiopia have emphasized the significant economic importance of the *Eucalyptus* tree [17]. Despite prioritizing *Eucalyptus* for higher income over cereal crops, Ethiopian farmers tend to overlook its negative environmental impacts, relying on its economic benefits to sustain rural households' income and food security, particularly in harsh conditions [20].

##### 3.1.2.2. Environmental Roles of Eucalyptus Tree in Ethiopia

Before the introduction of *Eucalyptus* to Ethiopia, fuelwood was primarily sourced from natural forests. With the advent of *Eucalyptus* plantations, the pressure on these forests has been significantly alleviated. These plantations have played a crucial role in meeting the growing demand for fuelwood and construction materials, thanks to the rapid growth and high-quality wood products of *Eucalyptus*. This shift has not only reduced deforestation but also contributed to biodiversity conservation by preserving native forest species [5].

In the Ethiopian Highlands, extensive planting of *Eucalyptus* trees has provided an alternative source of wood, thus diminishing the need to harvest timber from native forests and easing the strain on natural ecosystems. This change has led to reduced deforestation rates and offered some respite to remaining natural habitats. Moreover, *Eucalyptus* plantations can enhance local biodiversity by creating new habitats and preventing soil erosion [21, 22].

However, the introduction of *Eucalyptus* has not been without its drawbacks. Its tall height creates shading that suppresses the growth of underground plant species, and crops grown near *Eucalyptus* plantations often yield less than those grown further away. Despite these issues, the fast growth rate and high biomass storage capacity of *Eucalyptus* make it an effective tool for carbon sequestration, which helps combat global warming. The mixed outcomes underscore the need for careful management of *Eucalyptus* plantations to balance economic benefits with environmental sustainability [23–25].

### 3.2. Geographical Distribution of Eucalyptus in the World and East Africa

*Eucalyptus*, which extends across over seven billion hectares worldwide, is the most widely distributed tree species globally [26]. Brazil has the largest share, accounting for 42.11% of the total, while Thailand has the smallest, at 1.40% (Table 1).

### 3.3. History and Trends of Eucalyptus Plantation in Ethiopia

*Eucalyptus* was introduced to East Africa in the mid-19^th^ and early 20^th^ centuries by Europeans to address severe forest degradation and wood shortages. In Ethiopia, the introduction of *Eucalyptus* dates back to 1894/95, when Emperor Menelik II's advisors, French railway engineer Mondon-Vidaillet and British army officer O'Brien, brought the species to produce fuelwood and construction timber for the growing city of Addis Ababa. Initially, a small number of people began planting *Eucalyptus* around their homes in Addis Ababa. Due to its fast growth and adaptability to various environmental conditions, it quickly became one of the most widely planted tree species in the country. By the early 1970s, *Eucalyptus* plantations surrounding Addis Ababa covered approximately 15,000 ha, with around 76,000 ha in other parts of the country. Between 1975 and 1994, international donor agencies supported the establishment of new plantations in rural areas [17, 19].

The expansion of *Eucalyptus* plantations in Ethiopia accelerated during the 1990s and 2000s, reflecting a broader shift toward commercial and industrial forestry. This change was driven by the growing demand for fast-growing timber and fuelwood, as well as concerns about environmental degradation [28]. However, this rapid expansion has also raised environmental and ecological issues, such as soil depletion and reduced biodiversity [26].

The historical context of land conversion in Ethiopia is deeply rooted in the country's agricultural practices and socioeconomic development. Traditionally, Ethiopia's economy has been predominantly agrarian, with vast tracts of land dedicated to subsistence farming and pastoralism [29]. However, during the late 19^th^ and early 20^th^ centuries, under Emperor Menelik II, there was a significant shift toward modernizing agriculture, which included the introduction of *Eucalyptus* trees from Australia. *Eucalyptus* was promoted for its fast growth and utility in providing timber, fuelwood, and construction materials [13, 30]. Over time, as population pressure increased and deforestation escalated, *Eucalyptus* plantations expanded, especially around urban areas, due to their economic value and ability to grow on degraded lands. Today, *Eucalyptus* dominates many landscapes in Ethiopia, contributing to both economic development and environmental challenges, such as soil degradation and water depletion, reflecting a complex evolution of land use practices over the centuries [31].

Currently, there are 55 species of *Eucalyptus* grown in Ethiopia, with 5–10 species being widely cultivated [32]. The most commonly grown and dominant species include *Eucalyptus camaldulensis, Eucalyptus citriodora, Eucalyptus globulus, Eucalyptus regnans, Eucalyptus saligna, and Eucalyptus tereticornis. Eucalyptus globulus and Eucalyptus camaldulens*is are particularly dominant in the highlands of Ethiopia, where farmers cultivate them on small areas of land to produce a variety of wood. As forests and woodlands depleted, farmers increasingly turned to planting woodlots with fast-growing species like *Eucalyptus*. A study in North West Ethiopia revealed that by 2010, around 58% of planted forests were covered by *Eucalyptus*, with an estimated 500,000 ha dedicated to its cultivation [33]. The number of *Eucalyptus* planters continues to rise annually, underscoring the growing importance of this species in Ethiopia's forestry landscape.

### 3.4. The Expansion of Eucalyptus Plantation in Ethiopia

The conversion of forests and marginal lands into crop production areas, driven by population growth and improper resource use, has led to a scarcity of wood for fuel and construction. To address this issue, the replacement of native trees with *Eucalyptus* plantations has become increasingly popular [26]. *Eucalyptus* plantations are rapidly spreading, particularly in the highlands of Ethiopia, due to their high productivity and economic value [34]. However, this expansion must be closely monitored, as it can have detrimental effects on the environment, including altering native plant ecosystems and negatively impacting adjacent food crops through soil moisture depletion, nutrient competition, and shading [35].

Despite many farmers being aware of the negative environmental impacts of *Eucalyptus* trees, the demand for wood products, ease of cultivation, and high productivity of these trees have led to a significant increase in *Eucalyptus* plantations in Ethiopia. In recent years, *Eucalyptus* has become the most widely planted tree species, with many small-scale farmers establishing woodlot plantations. The growing demand for wood products and rising prices have further contributed to the rapid expansion of *Eucalyptus* plantations, a trend that has continued uninterrupted since the early 19^th^ century [36]. The spread of *Eucalyptus* plantations has also been facilitated by the simplicity of their cultivation and their high productivity [37].

The expansion of *Eucalyptus* plantations in Ethiopia has seen significant growth, particularly after the 1980s. This growth was largely driven by the rising prices of *Eucalyptus* poles, leading many households to plant over 100 red or white *Eucalyptus* trees each year. Between 1980 and 2010, the total plantation area steadily increased [38].

A study assessing the spatial extent of *Eucalyptus* plantations across three major regions in Ethiopia—Oromia, Amhara, and Southern Nations, Nationalities, and Peoples' Region—found that these plantations covered a total area of 68,000 ha [17]. Of this area, Oromia accounted for 43.68%, Amhara for 26.47%, and the Southern Nations, Nationalities, and Peoples' Region for 29.85%, with Oromia having the largest area of *Eucalyptus* plantations in the survey study carried out in 2023 [17].

In comparison to other tree species, *Eucalyptus* made up the largest portion (45%) of the spatial extent of plantations, followed by Cypress (42%), *Juniperus* (3%), Pine (2%), Grevillea (1%), and others (7%) in three Ethiopian regions (Oromia, Amhara, and Southern Nations, Nationalities, and Peoples' Region) in the study carried out in 2015 (Table 2).

### 3.5. The Causes of Eucalyptus Plantation Expansion in Ethiopia

A study on *Eucalyptus* tree farming expansion in Eza Wereda, West Guarage Zone, identified socioeconomic and environmental factors as the primary drivers of this expansion in Ethiopia [38]. Similarly, research conducted in the Lake Tana watershed of Northwest Ethiopia highlighted three main factors contributing to the spread of *Eucalyptus* farming: socioeconomic factors, the area's ecological characteristics, and the biological traits of the *Eucalyptus* tree [37]. These factors are discussed in detail below.

#### 3.5.1. Socioeconomic Factors

Population growth and land degradation have led to a significant rise in *Eucalyptus* tree farming in the West Guarage zone of Ethiopia. Farmers have adopted this practice to rehabilitate degraded areas, especially on uneven terrain. On average, each farmer plants about 61 *Eucalyptus* trees, resulting in a 70% increase in *Eucalyptus* plantation area over a 40-year period [38].

The expansion of *Eucalyptus* farming is further driven by the high demand for *Eucalyptus* products and the income potential from woodlot production. Between 1997 and 2008, the sharp increase in *Eucalyptus* plantations was fueled by favorable market prices for poles of various sizes [38]. A study in [37] reported that the Amhara Region exported 3,774,461 poles to Sudan through the Metema District Customs Office from 2006/07 to 2010/11, generating a total revenue of 12,214,327 USD.

In the Wegera district of Northern Ethiopia, approximately 61.66% of farmers converted their farmlands into *Eucalyptus* woodlots due to declining agricultural productivity and rising *Eucalyptus* prices. Similarly, another study in south-central Ethiopia found that nearly 88% of households planted *Eucalyptus* trees, citing their adaptability, rapid growth, resistance to livestock grazing, and substantial income potential from *Eucalyptus* wood products [39].

Several factors contribute to the expansion of *Eucalyptus* plantations in Ethiopia, including the increasing price of *Eucalyptus* wood, higher economic returns compared to annual crops, growing domestic and export markets, rising costs of agricultural inputs, and the demand for fuel energy and construction materials [2]. Additionally, social practices and holidays like Meskel and Arifa, which require substantial amounts of wood, further encourage the planting of Eucalyptus trees [38].

#### 3.5.2. The Ecological Characteristics of Ethiopia

*Eucalyptus* tree species can flourish in various agroecological zones, but the ecological conditions in Ethiopia are especially favorable for their growth. Research [18] indicates that the Koga watershed in the western Amhara Region offers an ideal environment for Eucalyptus plantations due to its medium altitude, optimal temperature, level terrain, fertile soil, and adequate annual rainfall.

#### 3.5.3. The Biological Properties of Eucalyptus Tree

The biological characteristics of Eucalyptus, including its rapid growth, adaptability to harsh environments, and resilience to stress and disease, make it a suitable option for various applications. Despite some limitations, such as its potential impact on surrounding crops and the high labor demands associated with its cultivation, a study in [10] found that 97% of farmers considered its benefits to outweigh its drawbacks. Additionally, 76% of the surveyed farmers expressed interest in planting Eucalyptus in the future.

### 3.6. The Impact of Eucalyptus Plantation on the Environment in Ethiopia

Although Eucalyptus provides significant socioeconomic and environmental benefits in Ethiopia, there is considerable debate among researchers and stakeholders about its overall impact. While some vehemently oppose its cultivation, others are strong proponents but still hold reservations [40]. This paper primarily examines the negative environmental effects of Eucalyptus plantations in Ethiopia, followed by a discussion of both the positive and negative aspects.

#### 3.6.1. Positive Impact of Eucalyptus Plantation on the Environment in Ethiopia

Eucalyptus trees are highly advantageous for Ethiopia due to their rapid growth and significant carbon sequestration capabilities [23]. They excel in various challenging environments, including degraded lands, swampy areas, unfertile soil, and dry regions. Eucalyptus can produce between 168 and 2900 kg of wood per hectare per year in the Ethiopian highlands, depending on soil conditions, stand age, and rotation cycle [26]. This efficiency in biomass production and carbon sequestration makes Eucalyptus a valuable asset for climate change mitigation and generating income through carbon trading [40].

However, while Eucalyptus trees offer immediate benefits in carbon sequestration and biomass, their long-term effectiveness is generally lower compared to slower-growing species like oak or sequoia, which sequester carbon over centuries [41]. Additionally, Eucalyptus forests often have reduced biodiversity and higher water usage, which can impact ecosystem resilience and sustainability [21]. Despite these challenges, Eucalyptus remains a key tool for addressing climate concerns and supporting economic activities in Ethiopia [42].

#### 3.6.2. Negative Impact of Eucalyptus Plantation on the Environment in Ethiopia

Eucalyptus plantations have faced criticism for their detrimental environmental impacts, including soil degradation, reduced water reserves, and the displacement of native vegetation. These plantations often cause water courses to dry up, suppress other plant species, lead to soil erosion, and negatively affect nutrient cycling and soil properties [27]. Researchers have also pointed to the allelopathic effects of Eucalyptus, which inhibit the growth of other plants and deplete soil nutrients. Additionally, the practice of Eucalyptus monoculture is blamed for threatening ecological stability and biodiversity [40].

##### 3.6.2.1. Impacts of Eucalyptus on Climate

Eucalyptus plantations can significantly affect the local climate. Their high evapo-transpiration rate results in a decrease in the groundwater table [26]. On average, a single Eucalyptus tree evaporates between 20 and 40 L of water per day [43]. This substantial loss of soil water in Eucalyptus plantations can lead to desertification and adversely impact local rainfall levels. However, distinguishing the specific effects of Eucalyptus plantations on regional rainfall and other climatic parameters, as compared to native forests, is challenging [19].

The impact of Eucalyptus on the microclimate at the local level is well-documented. When Eucalyptus plantations are established in *Acacia* forest sites, a shift in the microclimate occurs [26]. The extent of Eucalyptus's influence depends on the basal area of the tree cover. In shaded areas under Eucalyptus trees, mean air temperatures are lower and surface soil temperatures and air temperatures are reduced, while surface air humidity is higher compared to areas without tree cover. Generally, the larger the leaf area and the more horizontal the leaves, the greater the shading effect and higher the evapo-transpiration rate. The leaf size and orientation of Eucalyptus trees contribute significantly to this shading effect [19].

##### 3.6.2.2. Impacts of Eucalyptus on Water Resources

Eucalyptus plantations have several limitations, including reduced water sources and decreased water flow regulation on steep slope watersheds compared to natural forests [26]. Although runoff from an area depends on factors such as litter fall, vegetation cover, soil type, and climate, Eucalyptus plantations generate more runoff than grassland vegetation and natural forests, but less than cultivated land [44].

The environmental impacts of Eucalyptus plantations on water resources include changes in surface runoff, declines in soil moisture content, and reductions in groundwater recharge. According to a study conducted in the central highlands of Ethiopia [45], converting cultivated land to Eucalyptus plantation resulted in a 51.1% decrease in soil moisture content and a 48.9% decrease in spring water flow. Eucalyptus plantations also extract significant amounts of groundwater, which can lead to the drying up of rock reservoirs, alterations in spring water flow, and a decrease in the groundwater table. This was further demonstrated by research in the Koga watershed of the Amhara Region, Ethiopia [18], which found that Eucalyptus plantations near water sources could cause springs to dry up completely.

Additionally, a study in the Tropical Monsoon Climate of the Lake Tana Basin in Fogera, North West Ethiopia [46], showed that Eucalyptus plantations can decrease groundwater availability. The study observed an average daily fluctuation of 3.1 cm in the groundwater table due to the high water consumption by Eucalyptus trees. These trees have a transpiration rate that varies with solar irradiance and can reach up to 1.65 mm/hour during the day. During dry periods, Eucalyptus plantations can evaporate up to 2300 mm of water from groundwater—1400 mm more than in the absence of Eucalyptus trees. The average daily evapotranspiration is nearly twice the reference evapotranspiration and 2.5 times the actual rate under fallow agricultural fields [47].

Eucalyptus plantations can exacerbate water shortages due to their high water absorption rates compared to other tree species [26]. Their extensive root systems allow them to access water from deep soil layers, and their rapid evapotranspiration rates can lower the groundwater table, contributing to desertification. For example, *Eucalyptus grandis* consumes twice as much water as *Pinus patula* and up to five times more than *Podocarpus* and *Cupressus* trees of similar size during the dry season [48]. While Eucalyptus is not a significantly greater water user than other tree species and crops, its high biomass production leads to increased water consumption. Therefore, removing Eucalyptus forests can improve water yield and raise water tables in downstream areas [49].

##### 3.6.2.3. Impacts of Eucalyptus on Soil

Eucalyptus trees are known to deplete soil nutrients, leading to soil degradation and reduced crop yields. This issue arises primarily from their deep root systems, which drain water resources, combined with poor forestry practices like high planting densities and short crop rotations. A social survey study [46] has identified the decrease in soil fertility as one of the adverse ecological impacts of Eucalyptus. The tree's adaptability to various soil conditions enables it to extract nutrients beyond the root zones of other crops. Fast-growing and short-rotation Eucalyptus trees deplete soil nutrients more rapidly than slow-growing species.

When Eucalyptus is harvested, a significant portion of the major macronutrients stored in the above-ground biomass is removed. The concentration of nutrients such as nitrogen, phosphorus, potassium, calcium, and magnesium can vary among species [50].

Eucalyptus has an impact on soil moisture content. Measurements of moisture content near Eucalyptus stands during the dry season, at various soil depths, were found to be lower compared to those near *Croton macrostachyus* stands. However, soil pH, organic matter, exchangeable potassium, and bulk density were unaffected by Eucalyptus. Additionally, soils near Eucalyptus became highly hydrophobic after being air or oven dried, and dried Eucalyptus plant parts (leaves, bark, and roots) were slightly water-repellent. Another study observed differences in macronutrient concentrations with distance from Eucalyptus trees in the semihumid Ethiopian Highlands on the Lake Tana Plain. In this study, the concentration of macronutrients generally increased with distance from the Eucalyptus stands [20].

##### 3.6.2.4. Impacts of Eucalyptus on Crop

Eucalyptus trees require a significant amount of nutrients and water due to their rapid growth, which often leads to competition with adjacent crops for these resources. This competition can decrease soil fertility around Eucalyptus plantations, as the tree's deep roots extend into the soil to extract nutrients beyond the crops' feeding zone [51, 52].

Certain species of Eucalyptus can produce chemicals in their leaves or litter that hinder the germination or growth of other plant species. For example, *Eucalyptus camaldulensis*'*s* underground extracts impact the germination and early seedling growth of certain trees. The various solvent extracts of *Eucalyptus globulus* leaves have shown allelopathic effects on seedling germination and growth, inhibiting growth and reducing the germination rate. The inhibitory effect increases with extract concentration, with the highest concentration (5%–10%) causing the most significant inhibition. Studies have revealed that *Eucalyptus camaldulensis* has a notable inhibitory effect on tomato crops, particularly affecting shoot length, root length, leaf area index, and dry weight, as well as radicle/root length and germination efficiency. Consequently, Eucalyptus plantations can pose a potential threat to the vegetable industry under small-scale farming conditions [53, 54].

Eucalyptus is known for its allelopathic effects on crops, which are the direct and indirect impacts of allelochemical compounds produced by the plant. These compounds can either inhibit or stimulate the growth of other organisms. The production of biologically active molecules and their residues can influence the growth of both similar and dissimilar plants by negatively affecting key metabolic processes such as cell wall structure, cell division, enzyme activity, plant hormone balance, pollen tube germination, nutrient absorption, stomata function, photosynthesis, respiration, protein synthesis, pigmentation, and DNA/RNA structures [12, 53].

The cultivation of *Eucalyptus globulus* has been reported to cause various issues, including competition with cropland (74.5%), shading effects on crops (56.4%), and land use conflicts due to border effects (27.7%). According to a study, Eucalyptus trees can significantly reduce light intensity up to 5 m away to the west, up to 10 m away to the north at 12:30 p.m., and up to 15 m away to the east at 3:00 p.m. This reduction in light intensity is attributed to the dense root system of Eucalyptus trees, with 600 roots per square meter counted in the first 60 cm of the profile at a distance of 5 m from the tree. This results in a tenfold difference in biomass and yield of maize crops planted 20 m from Eucalyptus plantations. Therefore, the cultivation of Eucalyptus trees can significantly impact food security and agro-biodiversity [20, 45].

##### 3.6.2.5. Impacts of Eucalyptus on Biodiversity

Eucalyptus is known for its allelopathic effects, which inhibit the growth of underground plants by reducing soil nutrients necessary for undergrowth. This leads to soil degradation through increased erosion, nutrient depletion, and water loss, ultimately resulting in reduced biodiversity in the area [2]. Studies have shown that the presence of Eucalyptus plantations significantly hinders the root length of native species and negatively impacts the germination, root, and shoot growth of tomato seeds [1, 54]. Additionally, Eucalyptus trees have been found to decrease maize yield within 20 m of the trees and cause soil to become water-repellent [20]. Consequently, it is not advisable to plant tomatoes near Eucalyptus trees. Introducing nitrogen-fixing species could offer a solution by establishing a mixed stand with Eucalyptus and mitigating its negative effects [55].

## 4. Conclusion and Recommendation

The growing population in developing nations has increased the demand for wood, leading farmers to plant fast-growing, adaptable trees like Eucalyptus. This species is valued in Ethiopia for its diverse uses, including construction poles, firewood, charcoal, and fencing, providing additional income. Introduced by Emperor Menelik II in 1895 to address shortages of firewood and building materials in Addis Ababa, Eucalyptus plantations now span 506,000 ha in Ethiopia. While Eucalyptus plantations offer economic benefits such as high timber yields and enhanced land productivity, they also present environmental challenges. These include high water consumption that depletes surface and groundwater, reduces soil moisture, and releases chemicals that diminish soil fertility. Additionally, the high evaporation rate of Eucalyptus can alter the local microclimate, and its shedding and allelopathic effects may inhibit the growth of understory plants, reducing biodiversity and the availability of fodder for wildlife and livestock.

To address these environmental issues while maximizing economic benefits, effective management practices are essential. Strategies such as sustainable forestry, integrated land use planning, and the promotion of eco-friendly tree species can help balance economic gains with ecological health. Diversifying energy and construction resources, implementing land use planning, and offering alternative income opportunities are crucial steps. By reducing dependence on Eucalyptus through these measures, Ethiopia can mitigate environmental degradation. Cultivating fast-growing, native trees like highland bamboo offers both economic and ecological advantages, supporting a more sustainable approach to resource management and land use.

## Figures and Tables

**Table 1:** Area coverage of *Eucalyptus* plantation in some countries in the world.

No.	Country	Area coverage in hectares	Percent
1	Brazil	3,000,000	42.11
2	India	1,000,000	14.04
3	China	600,000	8.42
4	Portugal	550,000	7.72
5	South Africa	477,000	6.70
6	Spain	396,000	5.56.
7	Ethiopia	506,000	7.10
8	Morocco	200,000	2.81
9	Chile	170,000	2.39
10	Australia	125,000	1.75
11	Thailand	100,000	1.40
	Total	7,124,000	100.00

*Note:* Source: [27].

**Table 2:** Spatial extent of *Eucalyptus* plantation in relation to other industrial plantations in three Ethiopian regions.

No.	Tree species	Percentage coverage of *Eucalyptus* as compared to other tree species
1	*Eucalyptus*	45
2	*Cypress*	42
3	*Juniperus*	3
4	*Pine*	2
5	*Gravillea*	1
6	Others	7
	Total	45

*Note:* Source: [17].

## Data Availability

The data supporting this review are from previously reported studies and datasets, which have been cited.
